# Association between multimorbidity and hospitalization in older adults: systematic review and meta-analysis

**DOI:** 10.1093/ageing/afac155

**Published:** 2022-07-23

**Authors:** Luciana Pereira Rodrigues, Andréa Toledo de Oliveira Rezende, Felipe Mendes Delpino, Carolina Rodrigues Mendonça, Matias Noll, Bruno Pereira Nunes, Cesar de Oliviera, Erika Aparecida Silveira

**Affiliations:** Postgraduate Program in Health Sciences, School of Medicine, Federal University of Goiás, Goiânia, Brazil; Postgraduate Program in Health Sciences, School of Medicine, Federal University of Goiás, Goiânia, Brazil; Department of Nursing in Public Health, Federal University of Pelotas, Pelotas, Brazil; Postgraduate Program in Health Sciences, School of Medicine, Federal University of Goiás, Goiânia, Brazil; Postgraduate Program in Health Sciences, School of Medicine, Federal University of Goiás, Goiânia, Brazil; Federal Institute Goiano, Campus Ceres, Goiás, Brazil; Department of Sports Science and Clinical Biomechanics, University of Southern Denmark, Odense, Denmark; Postgraduate Program in Nursing, Federal University of Pelotas, Pelotas, Rio Grande do Sul, Brazil; Department of Epidemiology & Public Health, Institute of Epidemiology & Health Care, University College London, London, UK; Postgraduate Program in Health Sciences, School of Medicine, Federal University of Goiás, Goiânia, Brazil; Federal Institute Goiano, Campus Ceres, Goiás, Brazil

**Keywords:** multimorbidity, ageing, hospitalization, length of stay, readmission, older people

## Abstract

**Background:**

Multimorbidity is defined as the presence of multiple chronic conditions in the same individual. Multimorbidity is more prevalent in older adults and can lead to several adverse health outcomes.

**Methods:**

We systematically reviewed evidence from observational studies to verify the association between multimorbidity and hospitalization in older adults. Furthermore, we also aimed to identify whether it changes according to gender, advanced age, institutionalization, and wealth of the country of residence. We searched the PubMed, Embase and Scopus databases from December 2020 to April 2021. The analysed outcomes were as follows: hospitalization, length of stay and hospital readmission.

**Results:**

Of the 6,948 studies identified in the databases, 33 were included in this review. From the meta-analysis results, it was found that multimorbidity, regardless of the country’s wealth, was linked to hospitalization in older adults (OR = 2.52, CI 95% = 1.87–3.38). Both definitions of multimorbidity, ≥2 (OR = 2.35, 95% CI = 1.34–4.12) and ≥3 morbidities (OR = 2.52, 95% CI = 1.87–3.38), were associated with hospitalization. Regardless of gender, multimorbidity was associated with hospitalization (OR = 1.98, 95% CI = 1.67–2.34) and with readmission (OR = 1.07, 95% CI = 1.04–1.09). However, it was not possible to verify the association between multimorbidity and length of stay.

**Conclusions:**

Multimorbidity was linked to a higher hospitalization risk, and this risk was not affected by the country’s wealth and patient’s gender. Multimorbidity was also linked to a higher hospital readmission rate in older adults. PROSPERO Registration (Registration number: CRD42021229328).

## Key points

- Multimorbidity is associated with increased occurrence of hospitalizations and readmissions in older adults, regardless of the income level of the country.
- Most of the studies about the association between multimorbidity and hospitalization are concentrated in high-income countries.
- There is no gender difference in the association between multimorbidity and hospitalization outcomes.

## Introduction

Multimorbidity is defined as the presence of multiple chronic conditions in the same individual [1, 2]. Its prevalence ranges from 55 to 98% in older adults [3, 4], and increase with age [5, 6]. It is estimated that 80% of the population over 75 years have at least two chronic conditions [4]. Multimorbidity leads to low quality of life and increased functional disability compared with those without chronic diseases [7] and is accompanied by polypharmacy, higher number of hospitalizations and higher mortality risk [8, 9]. The number of hospitalizations may be associated with sociodemographic variables, especially socio-economic level and the co-occurrence of chronic conditions [10].

A Swiss study identified that the chance of being hospitalized and the length of stay among older adults with multimorbidity were two times higher and five to six times higher, respectively [11]. Previous research showed that multimorbidity increased the chance of hospital readmission [12]. Moreover, hospitalization in older adults increases the risk of death [13], loss of functionality [14], stress [15], mental health problems [16], cognitive impairment and social isolation [15]. Therefore, knowing the impact of multimorbidity on hospitalization is relevant.

Although multimorbidity in older adults is related to increased hospitalization, only one systematic review published in 2011 has focused on the subject [8], addressing the presence of multiple chronic conditions, rather than multimorbidity, as this term was inserted into the Medical Subject Headings (MeSH) thesaurus only in 2018. The aforementioned review evaluated the costs and use of health services in general, such as visits to the doctor’s office, use of medication and use of hospital services. Another systematic review on multimorbidity in older adults analysed its association with hospitalization outcomes; however, that was not the focus of the study [3]. Therefore, due to the paucity of evidence [3, 8], it is relevant to investigate the impact of multimorbidity on hospitalization in older adults.

Exploring this gap is relevant in the field of public health, geriatrics and gerontology, as it could assist health services to avoid hospitalizations, readmissions and greater length of stay of older adults with multimorbidity. In this sense, the main objective of this systematic review was to analyse the impact of multimorbidity on the occurrence of hospitalization in older adults. We also aimed to assess whether this impact is affected by gender, advanced age, institutionalization and country’s per capita income, and to identify the mean length of hospitalization and the occurrence of hospital readmission.

## Method

### Protocol and registration

This systematic review and meta-analysis were performed following the Preferred Reporting Items for Systematic Reviews and Meta-Analyses (PRISMA) methodology [17]. The Population, Exposure, Comparator and Outcome (PECO) structure recommended for systematic reviews [18]: ‘P’ (community-dwelling older adults), ‘E’ (multimorbidity), ‘C’ (associated factors) and ‘O’ (hospitalization). Our study was registered in PROSPERO (International Prospective Register of Systematic Reviews) (CRD42021229328). More details can be found in the systematic review protocol [19].

### Search strategy and eligibility criteria

The PubMed, Embase and Scopus databases were searched by two independent researchers from December 2020 to April 2021. The strategy used MeSH terms and relevant keywords on multimorbidity, hospitalization and older adults, aiming to cover all articles on this topic (Table S1, Supplementary Material). There were no restrictions on language and year of publication of the included studies, and articles published until 30 April 2021 were considered.

The following inclusion criteria were adopted for the studies: (i) cross-sectional, cohort and case–control methodologies; (ii) use of the definition of multimorbidity as ≥2 and/or ≥ 3 chronic conditions; (iii) outcomes that included length of stay or number of hospitalizations or readmissions and (iv) participants aged 60 years or older. We chose to use the age range ≥60 years to include both the predominant definition of ‘older adult’ in developed countries (i.e. 65 years or older) [20] and that in developing countries (i.e. 60 years or older) [21].

There are different ways to operationalize and define multimorbidity. Although Fortin *et al.* [22] suggest the use of the term to refer to the co-occurrence of three or more chronic conditions in the same individual, to better identify individuals who need more health care, i.e. older adults, the prevalent definition is the co-occurrence of two or more chronic conditions. In a systematic review that included seventy studies, the difference in prevalence between multimorbidity ≥2 and ≥3 chronic conditions was 12.9% [23]. Thus, this review included studies that defined multimorbidity as the co-occurrence of ≥2 and ≥3 chronic conditions.

Review articles, ecological studies, case reports or series, incomplete data as well as duplicate data and unavailable data even after contacting the authors of the studies were excluded. Also excluded were studies that analysed a baseline disease or index (e.g. cancer, heart disease, depression), studies that used other definitions of multimorbidity (clusters, latent class analysis, Charlson Comorbidity Index, Cumulative Disease Rating Scale, etc.) and studies that included indigenous populations or that addressed other age groups (except if stratified to extract only the data related to older adults). Finally, conference abstracts, theses and dissertations (grey literature) were also excluded from this systematic review.

### Review process

**Figure 1:**
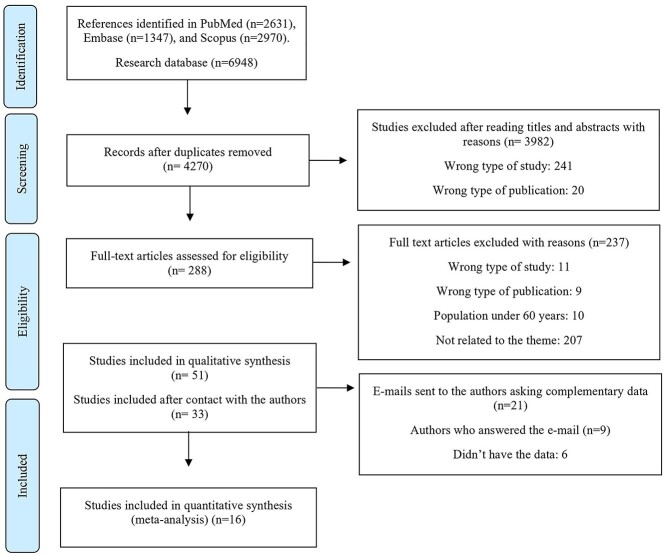
Flow diagram of search process.

Duplicate studies were excluded using the Mendeley software. Then, two independent authors (ATOR and LPR) performed the reading of titles and abstracts of all selected articles using the Rayyan software [24]. After that, the selected studies were read in full and evaluated according to the eligibility criteria. Since 21 studies did not present sufficient data considered important for the research, one of the researchers contacted the authors directly to obtain such information [6, 25–44]. Disagreements were discussed and resolved by a third senior reviewer (SAE). All researchers involved in this review process have experience in conducting systematic reviews and underwent training to perform the selection of studies according to the eligibility criteria. The PRISMA flowchart of the review process is shown in Figure 1.

### Data extraction and quality assessment

Data extraction was performed using a standardized form prepared by the authors, containing: author/year/location (city/country); type of study and population (number of participants, age range, follow-up time, whether institutionalized or not), definition and occurrence of multimorbidity (≥2 and/or ≥3 chronic conditions, number of chronic conditions considered, incidence or prevalence), length of stay and number of hospitalizations and readmissions (definition, prevalence and confidence interval). The measures of impact of multimorbidity on the outcomes analysed were prevalence or odds ratio (OR) with their 95% confidence intervals (CI). The data extraction Table was divided using the World Bank categorization [45], which classifies each country into high, middle and low income according to its per capita income (Table 1).

**Table 1:** Summary of studies that associated multimorbidity and hospitalization in the high-income countries

Author year location	Study design population^a^	Multimorbidity definition/occurrence	Hospitalization/length of stay/readmission definition/occurrence	Impact of multimorbidity on hospitalizations/length of stay/readmission
**Cohort/hospitalization**				
Buja *et al.* 2020 Vicenza, Italia	Cohort 1,975 participants ≥65 years 1 year follow-up community	**MM:** ≥2CC List of 14CC **Incidence**: Overall: NR 2CC: 10.06% 3CC: 21.6%	Hospital discharge records in 2013 to identify patients who experienced any of the following: at least one hospital admission, at least two hospital admissions and total number of hospital admissions **Hospitalization incidence**: NR	Regression models adjusted for age/gender **Hospitalization by multimorbidity (OR [95% CI])**^b^**:** **At least 1 admission per 3CC:** 1.32 [0.91–1.93] **At least 2 admissions per 3CC**: 1.37 [0.78–2.52] **Hospitalization per 3CC (IRR [CI]:** 1.35 [1.00–1.84]
Chamberlain *et al*. 2019 Minnesota, USA	Cohort 16,267 participants 60–89 years 11 years follow-up community	**MM:** ≥3CC List of 18CC **Incidence**: Overall: 35.63% 60–69 years: 41.9% 70–79 years: 39.9% 80–89 years: 18.2%	Hospitalizations for any cause were obtained from January 1, 2006, through December 31, 2016 **Hospitalizations (median number per person)**: 60–69 years: 1 70–79 years: 2 80–89 years: 3	Regression models adjusted for age, sex, race, ethnicity, education and marital status **Hospitalization by multimorbidity (HR [95% CI]):** 60–69 years: 1.78 [1.64–1.94] 70–79 years: 1.65 [1.54–1.78] 80–89 years: 1.64 [1.46–1.83]
Halonen *et al*. 2019 Tampere, Finland	Cohort 2,862 participants ≥90 years Follow-up waves: 2001, 2003, 2007 and 2010 community/institutionalized	**MM: ≥**2CC List of 9CC **Incidence**: Overall: NR 2CC: 27.5% 3CC: 24.2% **Women** 2CC: 28.2% 3CC: 24.9% **Men** 2CC: 26.5% 3CC: 23.0%	Long-term care: an approval for LTC admission from the municipal authorities or being at least 90 days in a residential home, service home with 24-h assistance or inpatient ward of a health centre or hospital **Hospitalization incidence:** NR	Regression models adjusted for age, year of entry, occupational status and living arrangements **Hospitalization by multimorbidity (SHR [95% CI]):** **Women** 2CC: 1.43 [0.98–2.08] 3CC: 1.64 [1.12–2.40] **Men** 2CC: 1.52[0.82–2.78] 3CC: 1.57 [0.83–3.00]
Wagner *et al*. 2019 Washington, USA	Cohort Older adults’ sample: NR 66 years (mean age, SD: 14.5) 5 years follow-up community	**MM:** ≥2CC List of 9CC **Incidence:** Overall: NR 2CC: 26.2% ≥3CC: 23.2%	Inpatient hospital admission in the last 30 days of life **Hospitalization incidence:** NR	Regression models adjusted for racial, minority status, level of education, age at death, gender, facility providing care, and type of healthcare insurance **Hospitalization by multimorbidity (OR [95% CI]):** 2CC: 1.75 [1.61–1.90] ≥3CC: 2.80 [2.57–3.05]
Ensrud *et al*. 2018 USA	Cohort 1,701 men participants ≥65 years 1 year follow-up community-dwelling	**MM**: ≥2CC List of 31CC **Incidence:** Overall: NR 2–4CC: 42.7%	Hospital stays and inpatient facility days for the 12-month period **Hospitalization incidence**: 18.5%	Regression models adjusted for marital status, health status, depressive symptoms, physical activity **Hospitalization by multimorbidity:** 2–4CC: 17.9%
Collerton *et al*. 2016 Newcastle, England	Cohort 710 participants ≥85 years 17-month follow-up institutionalized	**MM:** ≥ 2CC List of 20CC **Incidence**: Overall: 92.7%	Data on overnight hospital admissions in a timeframe of 12 months **Hospitalization incidence**: NR	Kruskal-Wallis tests **Hospitalization by multimorbidity:** Any overnight hospital admission: 34.1% Any ‘Day Hospital’ attendance: 7.6%
Gruneir *et al*. 2016 Ontario, Canada	Cohort 1,634,390 participants ≥65 years 1 year follow-up community	**MM:** ≥2CC List of 16CC **Incidence**: Overall: 48.67% 2CC: 27.4% 3CC: 45.7%	Any unplanned hospitalization within a year: **Hospitalization incidence:** 5.6%	Regression models **Hospitalization by multimorbidity:** 2CC: 10.34% 3CC: 13.87%
**Cohort/readmission**				
Shebeshi *et al*. 2020 Australia	Cohort 2,056 women participants 75–95 years community	**MM**: ≥2CC List of 6CC **Incidence:** NR	Readmission 28 days post-discharge **Readmission incidence:** 17.7%	Regression models **Readmission by multimorbidity (HR [95% CI]):** >2CC: 1.21 [0.79–1.83]
Aubert *et al*. 2019 Switzerland/USA/ Israel	Cohort Older adults’ sample: NR 64 years (mean age, SD: 52–76) 1 year follow-up community/nursing home	**MM:** ≥2CC 18 body system categories **Incidence:** Overall: 85.8%	30-day all-cause readmission **Readmission (median [interquartile range]):** 0 [0–2] **Length of stay (median [interquartile range]):** 4 [3–8]	Regression models **Readmission by multimorbidity (OR [95%CI]):** 2CC: 1 [1.0] 3CC: 1.04 [0.98–1.10]
**Cohort/*length of stay***				
Aubert *et al*. 2019 Bern, Lausanne, Geneva, Switzerland	Cohort Older adults’ sample: NR 68 years (mean age, SD: 56–78) 1 year follow-up community	**MM:** ≥2CC List of 18 body system **Incidence:** Overall: 79.3% (median: 68 years)	Length of stay: number of days from hospital admission to hospital discharge any inpatient ward of the same hospital within 30 days following hospital discharge **Length of stay incidence (median [interquartile range]):** 5 [3–8]	Regression models **Length of stay (OR [95%CI]):** 1 [1]
**Cohort/*hospitalization, length of stay and readmission***				
Navickas *et al*. 2015 Lithuania	Cohort 271,866 participants ≥65 years 2,5 years follow-up community	**MM:** ≥2CC List of 32CC **Incidence**: Overall: 2CC: 43.24% 3CC: 54.12% **65–74 years** 2CC: 22.75% **75–84 years** 2CC: 15.37% **85+ years** 2CC: 5.13%	Hospitalizations, readmission within 30 days and length of stay **Hospitalization incidence**: NR	Regression models **Hospitalization, length of stay and readmission by multimorbidity (proportion[mean]):** **Hospitalization** 65–74 years: 0.17 [0.54] 75–84 years: 0.21 [0.55] 85+ years: 0.23 [0.56] **Length of stay (days):** 65–74 years: 0.84 [9.82] 75–84 years: 0.21 [10.84] 85+ years: 9.88 [7.99] **Readmission:** 65–74 years: 0.15 [0.54] 75–84 years: 13 [0.47] 85+ years: 0.09 [0.33]
**Cross-sectional/hospitalization**				
Kim *et al*. 2020 South Korea	Cross-sectional Older adults’ sample: ≥65 years community	**MM:** ≥2CC List of 28CC **Prevalence:** NR	Inpatient visits over the past one year **Hospitalization prevalence:** NR	Regression models **Hospitalization by multimorbidity (OR [95%IC])**^b^**:** 1.53 [1.36–1.73]
Mitsutake *et al*. 2019 Tokyo, Japan	Cross-sectional 1,311,116 participants ≥75 years community	**MM**: ≥2CC and ≥ 3CC List of 21CC **Prevalence**: Overall: NR ≥2CC: 80.2% ≥3CC: 65%	Number of hospital admissions during September 1, 2013, and August 31, 2014 **Hospitalization prevalence:** NR	Regression models adjusted for age, sex and household income. **Hospitalization (number) by multimorbidity per 3CC (OR [95%CI]**: 1: 1.50 [1.47–1.54] 2: 1.86 [1.79–1.93] ≥3: 2.72 [2.58–2.87]
Gandhi *et al*. 2018 Hawaii, USA	Cross-sectional 84,212 participants ≥65 years community	**MM**: ≥2CC List of 15CC **Prevalence**: Overall: NR 2–3CC: 36.3%	Having one or more claims for an inpatient admission at any given time in 2012 **Hospitalization prevalence:** NR	Regression models adjusted for age, gender, dual eligibility, residential area **Hospitalization by multimorbidity (OR [95%CI]):** 2–3CC: 4.81 [4.31–5.37]
Rodrigues *et al*. 2018 Portugal (the mainland, Azores, and Madeira)	Cross-sectional 2,393 participants ≥ 65 years community	**MM**: ≥2CC List of 12CC **Prevalence:** Overall: 78.3%	Hospitalization in the previous 12 month **Hospitalization prevalence:** 25.8%	Regression models adjusted for age and gender: **Hospitalization by multimorbidity (OR [95%CI])**^b^**:** 1.91 [1.39–2.62]
Wolff *et al*. 2015 USA	Cross-sectional 1,217,103 participants ≥65 years community	**MM:** ≥2CC and ≥ 3CC List of 16CC **Prevalence:** Overall: NR 2CC: 65% 3CC: 43%	Hospitalizations for ambulatory care sensitive conditions within a year **Hospitalization prevalence:** NR	Regression models **Hospitalization by multimorbidity (OR [95% CI]):** 2CC: 18.10 [15.79–20.76] 3CC: 36.43 [31.81–41.73]
Nägga *et al*. 2012 Linkoping, Sweden	Cross-sectional 496 participants ≥85 years community	**MM:** ≥2CC List of 14CC **Prevalence**: Overall: 68%	Hospitalization over the preceding 12 months **Hospitalization prevalence:** NR	Regression models **Hospitalization by multimorbidity (OR [95%CI])**: 2.1 [1.3–2.5] *p* = 0.002
Glynn *et al*. 2011 Ireland	Cross-sectional 2,000 participants ≥60 years community	**MM:** ≥2CC List of 9 system ICPC-2 coding of chronic diseases **Prevalence**: **Women** Overall: 60.23% 60–69 years: 69.4% 70–79 years: 84.1% 80 + years: 89.0% **Men** Overall: 60.65% 60–69 years: 66.2% 70–79 years: 82.4% 80 + years: 88.2%	Hospital admission in the previous 12 months **Hospitalization prevalence**: NR	Regression models adjusted for gender, free medical care eligibility **Hospitalization by Multimorbidity (OR [95% CI]):** 2CC: 1.86 [1.18–2.94] 3CC: 2.12 [1.33–3.38]
**Cross-sectional/*length of stay***				
Picco *et al*. 2016 Singapore	Cross-sectional 2,565 participants ≥60 years community	**MM**: ≥2CC List of 10CC **Prevalence**: Overall: 51.5% 60–74 years: 47.2% 75–84 years: 65.3% 85+ years: 59.8%	Inpatient care during the three-month period prior to the interview **Length of stay prevalence:** NR	Regression models adjusted for age, gender, ethnicity, marital status, education and employment status **Length of stay by multimorbidity (mean [SE]):** ≥2CC: 2.7 [0.6]
Wister *et al*. 2016 Canada/Australia	Cross-sectional 9,886 participants (Canada); 1,858 (Australia) ≥65 years community	**MM:** ≥2CC List of 7CC **Prevalence**: NR	Length of stay in the last year **Length of stay prevalence:** NR	Regression models adjusted for age, gender and country. All coefficients were adjusted for marital-status, foreign born status and education level **Length of stay (OR [95 %CI]):** **Canada** **Women** 65–74 years: 1.54 [1.40, 1.70] 75 + years: 1.44 [1.32,1.58] **Men** 65–74 years: 1.46 [1.32,1.61] 75+ years: 1.41 [1.28,1.56] **Australia** **Women** 65–74 years: 1.35 [1.12,1.63] 75 + years: 1.31 [1.06,1.61] **Men** 65–74: 1.60 [1.31,1.96] 75+ years: 1.52 [1.23,1.88]
**Cross-sectional/*readmission***				
Conner *et al*. 2019 USA	Cross-sectional 2,375,331 participants ≥65 years community	**MM:** ≥2CC List of 25CC **Prevalence**: Overall: 15% 2CC: 5.7% 3CC: 9.3%	30-day all-cause unplanned hospital readmissions **Prevalence (% [95%CI]**): 11.9 [11.7–12.0]	Regression models **Readmission by Multimorbidity (OR [95%CI]):** 2CC: 1.06 [1.03–1.10] 3CC: 1.08 [1.05–1.12]
Lochner *et al*. 2013 USA	Cross-sectional 31,6 million ≥65 years community	**MCC:** ≥2CC List of 15CC **Prevalence**: Overall: NR ≥2CC: 67,3%	An admission to an acute care hospital for any cause within 30 days **Readmission prevalence:** NR	Statistical analysis: NR **Readmission by multimorbidity:** 2–3CC: 10.3%
**Cross-sectional/*hospitalization and length of stay***				
Bähler *et al*. 2015 Switzerland	Cross-sectional 229,493 participants ≥65 years community	**MM:** ≥2CC List of 22CC **Prevalence**: Overall: NR 2CC: 76.6%	Number of hospitalizations, if any, and the mean length of hospital stay in a year **Hospitalization prevalence:** NR	Regression models **Hospitalization and length of stay (mean number [median])** Hospitalizations: 1.5 [0.9] Length of stay: 14.3 [21.0]

*Abbreviations:* CC: chronic conditions, CI: confidence interval, HR: hazard ratio, MM: multimorbidity, OR: odds ratio, SE: standard ratio, SHR: sub hazard ratio, Std Error: standard error

NR: not reported

^a^The sample included in the Table was the age group 60 years or older

^b^OR sent by the author after email request.

The risk of bias during the process of selection of studies was assessed using the Downs & Black Scale [46]. However, only the items related to observational studies [1–3, 5–12, 17, 19, 20, 24, 25] were applied [47]. Studies with scores higher than 70% were considered as having a low risk of bias [46].

The Grading of Recommendations, Assessment, Development and Evaluations (GRADE) was used to evaluate the quality of the evidence [48]. In each study, the quality was attributed to the following grades: high quality (four filled circles), moderate quality (three filled circles), low quality (two filled circles) or very low quality (one filled circle). Observational studies begin the evaluation with two circles and some items may compromise the quality of evidence, such as risk of bias, imprecision, inconsistency, indirectness and publication bias. In contrast, the quality of evidence may increase (additional filled circles) when the effects are relevant, and all biases underestimate the effect or when a dose–response gradient is present [48].

### Statistical analysis

The impact of multimorbidity on the occurrence of hospitalization, readmission and length of stay in older adults was summarized, as well as the meta-analysis of the mean length of stay and the occurrence of hospital readmission. For both, meta-analyses, i.e. the impact of multimorbidity on the occurrence of hospitalization and the analysis of the occurrence of hospital readmission, a random effects model forest plot was built using OR results and their respective 95% CIs. The combined results were stratified according to socio-economic level (upper middle-income and lower middle-income countries).

Studies that reported data as hazard ratio (HR) or relative risk (RR) were converted into ORs, using the following formula: OR = ((1 – *p*) ^*^ RR)/(1 – RR ^*^p), where RR or HR is the relative risk or hazard ratio, respectively, OR is the odds ratio and *p* is the rate of the event in the control group [49]. A random effects model was used to perform the analyses. The results were reported as OR and their respective 95% CI. When possible, we considered the adjusted values of the studies for inclusion in the meta-analysis. Statistical analyses were conducted with the R language, version 4.1.0, using the miniMeta package.

### Heterogeneity assessment and additional analyses

The Higgins I^2^ statistic was calculated to estimate statistical heterogeneity among studies, considering values above 50% and *p* < 0.05 as high heterogeneity [50], and forest plots were constructed to graphically display effect sizes among studies [51]. Publication bias was assessed using funnel plots [52] and Egger’s test of funnel plot asymmetry [53].

## Results

This review identified 6,948 articles, and after exclusion of duplicates, 4,270 articles remained. Of these, after applying the eligibility criteria, 288 were selected for full-text reading, after which a total of 51 articles were selected. However, as 21 of these required additional data [6, 25–44], we contacted the respective authors, and obtained eight responses [6, 29, 30, 37, 40, 44, 46, 47]. Thus, 33 articles were included in this systematic review [11, 12, 27, 34, 54–82], of which 16 were included [12, 27, 34, 54, 56, 58–60, 64, 68, 69, 71, 74, 77–79] in the meta-analysis (Figure 1).

There were 23 studies [11, 12, 27, 34, 54–65, 73, 76–81] from high- and 10 studies [66–72, 74, 75, 82] from upper middle- and lower-income countries. Twenty-one studies (63.6%) were cross-sectional [11, 27, 34, 56–61, 63–65, 67–72, 74, 75, 82] and 12 cohorts [12, 54, 55, 62, 66, 73, 76–81], with a follow-up ranging from 1 to 11 years. The number of individuals in the studies ranged from 496 to 31.6 million [59, 65] (Tables 1 and 2).

**Table 2:** Summary of studies that associated multimorbidity and hospitalization in the upper middle- and lower middle-income countries

Author year location	Study design population^a^	Multimorbidity definition definition/occurrence	Hospitalization/length of stay/readmission definition/occurrence	Impact of multimorbidity on hospitalizations
**Upper middle income**				
**Cohort/hospitalization and length of stay**				
Lai *et al*. 2019 Hong Kong, China	Cohort 94,225 participants ≥60 years 9 years follow-up community	**MM**: ≥2CC List of 40CC **Incidence:** Overall per 2CC: 83.85% Overall per 3CC: 89.83% **60–64 years** 2CC: 6.8%/≥3CC: 5.0% **65–69 years** 2CC: 11.2%/≥3CC: 5.0% **70–74 years** 2CC: 16.6%/≥3CC: 10.0% **75–79 years** 2CC: 18.7%/≥3CC: 20.5% **80–84 years** 2CC: 15.4%/≥3CC: 19.3% **85–89 years** 2CC: 9.2%/≥3CC: 12.2% **90–94 years** 2CC: 4.5%/≥3CC: 5.0% **95–99 years** 2CC: 1.1%/≥3CC: 1.2% **100+ years** 2CC: 0.3/≥3CC: 0.2%	Number of annual hospital admissions, and annual number of length of stay **Hospitalization incidence:** NR **Length of stay incidence:** NR	Regression models adjusted for sex, comprehensive social security assistance recipient status, elderly home residential status, and number of days survived **Hospitalization by multimorbidity follow-year 9 (RR [95% CI])** **65–74 years** 2CC: 0.54 [0.48–0.61] ≥3CC: 0.64 [0.54–0.75] **75+ years** 2CC: 0.40 [0.36–0.45] ≥3CC: 0.53 [0.46–0.62] **Length of stay by multimorbidity follow-year 9:** **65–74 years** 2CC: 1.70 [1.51–1.92] ≥3CC: 2.31 [1.92–2.77] **75+ years** 2CC: 2.08 [1.83–2.35] ≥3CC: 2.26 [1.91–2.67]
**Cross-sectional/*hospitalization***				
Garcia-Ramirez *et al*. 2020 Colombia	Cross-sectional 23,694 participants ≥60 years community	**MM:** ≥2CC List of 7CC **Prevalence**: Overall: 45.63%	Hospitalizations in the last year **Hospitalization prevalence**: 12.9%	Regression models **Hospitalization by multimorbidity (OR [SE]):** 2.59, 0.432
Li *et al*. 2020 China	Cross-sectional 5,166 participants ≥65 years community-dwelling	**MM**: ≥2CC List of 14CC **Prevalence**: Overall: NR 65–69 years: 39.5% 70–74 years: 28.2% 75–79 years: 17.4% 80+ years: 14.9%	Inpatient visits in the last year **Hospitalization prevalence**: NR	Regression models **Hospitalization by multimorbidity**: 24.6% Truncated negative binomial model **Number of hospitalization (mean [SD]):** 1.63 [1.14]
Cheung *et al*. 2019 Hong Kong, China	Cross-sectional 2,618 participants ≥60 years community-dwelling	**MM**: ≥2CC List of 7CC **Prevalence**: Overall: 41.8% 2CC: NR 3CC: 10.4%	Hospital admission in the past 12 months **Hospitalization prevalence**: 23.6%	Regression models adjusted for age, gender, marital status, education and living arrangement **Hospitalization by multimorbidity (OR [95%CI])**: Overall: 1.25 [1.04–1.51] 2 CC: 1.74 [1.26–2.40] 3CC: 2.82 [1.93–4. 12]
Wang *et al*. 2018 Shenzhen City, China	Cross-sectional 2,603 participants ≥60 years community-dwelling	**MM**: ≥2CC List of 17CC **Prevalence**: Overall: 45.06%	Annual hospitalization **Hospitalization prevalence:** 10.50%	Regression models **Hospitalization by Multimorbidity:** 15.9%
Nunes *et al*. 2017 Bagé, Brazil	Cross-sectional 1,593 participants ≥60 years community	**MM** ≥ 2CC and ≥ 3CC List of 17CC **Prevalence (% [95%CI]):** Overall: NR 2CC: 81.3% [79.3–83.3] 3CC: 64.0% [61.5–66.4]	Hospitalization in the last year **Hospitalization prevalence (% [95%CI]):** 17.7 [15.8–19.6]	Regression models adjusted for gender, age, skin colour, marital status, economic classification and education **Hospitalization by multimorbidity (PR [95%CI]):** ≥2CC: 1.75 (1.21–2.51) ≥3CC: 1.94 (1.46–2.56)
Nunes *et al*. 2015 Bagé, Brazil	cross-sectional 1,593 participants ≥60 years community	**MM ≥ 2 and ≥ 3** **List of 17CC Prevalence (% [95%CI]):** **Overall:** NR **2CC: 81.3% [79.3–83.3]** **3CC: 64.0% [61.5–66.4]**	Hospitalization in the 12 months Hospitalization prevalence: NR	Hospitalization by multimorbidity (% [95%CI]): ≥2CC: 88.2 [84.3–92.2] ≥3CC: 76.5 [71.2–81.7]
**Lower middle income**				
**Cross-sectional/hospitalization**				
Pati *et al*. 2020 Odisha, India	Cross-sectional Older adults’ sample: NR ≥60 years community	**MM**: ≥2CC List of 21CC **Prevalence (% [95%CI])**: Overall: NR 60–69 years: 6.9 [28.1–45.8] ≥70 years: 4.4 [33.0–55.8]	Inpatient admissions in the last 12 months **Hospitalization prevalence:** NR	Regression models adjusted for gender, ethnicity, socio-economic status, highest education, marital status **Hospitalization (IRR [95%CI]):** **Public** 60–69 years: 3.84 [2.23–6.59] ≥70 years: 2.98 [1.62–5.49] **Private** 60–69 years: 1.68 [0.44–6.46] ≥70 years: 1.96 [0.55–6.96]
Mini *et al*. 2017 India	Cross-sectional 9,852 participants ≥60 years community	**MM:** ≥2CC List of 12CC **Prevalence**: Overall: 30.7%	Hospitalization in the past 1 year **Hospitalization prevalence:** NR	Regression models adjusted for age-sex **Hospitalization by multimorbidity (OR [95%CI])**: 2.32 [1.82–2.95]
Marthias *et al*. 2021 Indonesia	Cross-sectional 2,712 participants ≥61 years community	**MM**: ≥2CC List of 14CC **Prevalence (% [95%CI]):** Overall: NR 61–70 years: 21.9 [19.2 to 24.8] 71 + years: 25.2 [20.6 to 30.4]	Inpatient visits in the last 12 months **Hospitalization prevalence:** NR	Regression models **Hospitalization by multimorbidity (OR [95%CI])** Any visit 61–70 years: 1.23 [0.91–1.65] 71 + years: 1.42 [1.02–2.00] **Number of visits (IRR [95% CI]):** 61–70 years: 1.23 [0.88–1.72] 71 + years: 1.53 [1.01–2.32]

*Abbreviations:* CC: chronic conditions, CI: confidence interval, HR: hazard ratio, IRR: incidence rate ratio, MM: multimorbidity, OR: odds ratio, SE: standard ratio, SHR: sub hazard ratio, Std Error: standard error.

NR: not reported

^a^The sample included in the table was the age group 60 years or older

Twenty-three articles considered multimorbidity as the presence of two or more chronic conditions [11, 12, 27, 34, 55, 57, 59–65, 67, 68, 70, 72–75, 77, 81, 82], one article as three or more [79] and nine used both definitions [54, 56, 58, 66, 69, 71, 76, 78, 80]. Considering the outcomes, 23 studies evaluated the association of multimorbidity with hospitalization [27, 34, 54, 56–60, 62, 68–76, 78–80, 82], four with readmission [12, 64, 65, 77] and three with length of stay [61, 63, 81]. Two articles evaluated two outcomes: hospitalization and length of stay [11, 66], and one article analysed hospitalization, length of stay and readmission [55] (Tables 1 and 2).

The list of diseases ranged from 6 [77] to 40 [66] diseases since 54% of the studies used 10–20 diseases [27, 57, 59, 61, 65, 68, 70, 71, 73, 74, 76, 78, 79, 82], 25% used 21 or more diseases [11, 34, 55, 56, 62, 66, 72], 21% used 6–9 diseases [54, 63, 67, 69, 77, 80] and five studies did not report [12, 58, 60, 64, 81]. The five most frequently diseases were: diabetes (in 27 of the 33 studies), hypertension (in 27 of the 33 studies), cancer (in 26 of the 33 studies), coronary heart disease (in 25 of the 33 studies) and stroke (in 23 of the 33 studies).

Administrative data (11 studies) [11, 55, 57, 58, 62, 64, 65, 76–78], self-report (15 studies) [19, 34, 59, 61, 63, 67–72, 74, 75, 80, 82] and medical report (7 studies) [12, 54, 60, 66, 73, 79, 81] were the multimorbidity data sources reported. Among these, self-report was the most used; followed by administrative data and medical reports. With regards to the measures adopted to assess multimorbidity, this review found: Disease Count (30 articles), ACG system (2 articles) [58, 78] and Latent Class Analysis (1 article) [41].

The scores obtained in the Downs & Black Scale ranged from 53.8% to 100% (Appendix 1), and 30 studies included in the review had scores above 70%, indicating a low risk of bias. Regarding the evaluation of the methodological quality through GRADE, 8 studies presented moderate quality [12, 61–63, 69, 71, 72, 76], 20 presented low quality [11, 27, 54, 55, 57–60, 66–68, 70, 74, 75, 78, 80, 81] and 5 presented very low quality [34, 56, 64, 65, 79]. Most studies (29; 87.9%) declared no conflict of interest and 24 (72.7%) reported ethical approval. Related to the funnel plot analysis, the Egger test showed no asymmetry across the studies (*p* = 0.1) (Appendix 2).

**Figure 2:**
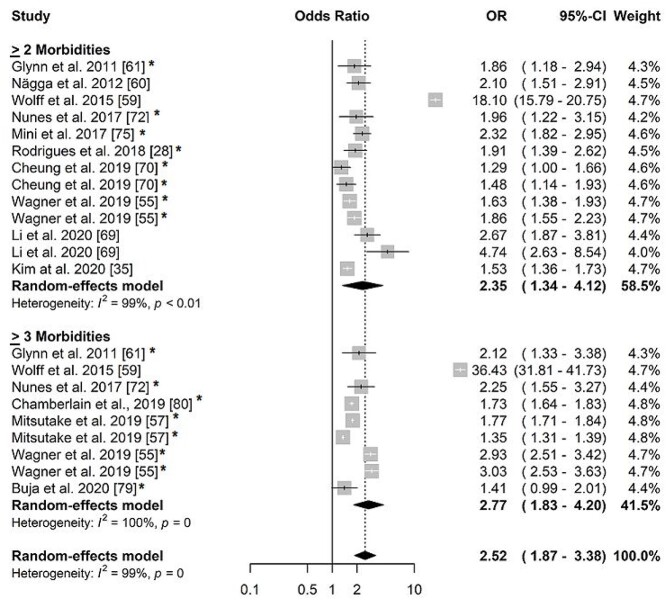
Forest plot of odds ratio of the association between multimorbidity and hospitalization in older adults stratified by income.

**Figure 3:**
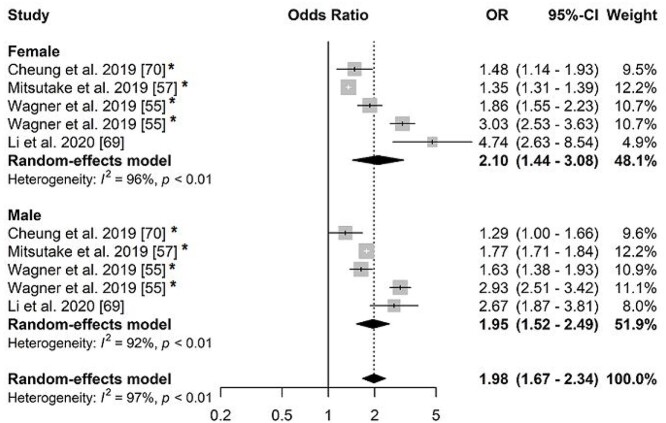
Forest plot of odds ratio of the association between multimorbidity and hospitalization in older adults stratified by ≥2 and ≥3 morbidities.

For the 16 articles included in the meta-analysis [12, 27, 34, 54, 56, 58–60, 64, 68, 69, 71, 74, 77–79] the OR of the association between multimorbidity and hospitalization according to the country’s income was 2.52 (1.87–3.38) (Figure 2, Appendix 2). When stratified by per capita income, only one study [83] did not show an association between multimorbidity and hospitalization. When stratified by two and three chronic conditions, only one article [78] found no significant association between multimorbidity ≥3 and hospitalization (Figure 3). In the meta-analysis of the association between hospitalization and multimorbidity ≥2 chronic conditions, the OR was 2.35 (95%CI: 1.34; 4.12, *I*^2^: 99%), while for ≥3 chronic conditions, it was 2.77 (95%CI: 1.83–4.20, *I*^2^: 100%) (Figure 3).

Focusing on length of stay, six studies [11, 55, 61, 63, 66, 81] evaluated this outcome. However, it was not possible to perform a meta-analysis due to insufficient data for pooling. Three studies [11, 55, 61] used the mean length of stay associated with multimorbidity ≥2CC ranging from 2.7 [61] to 14.3 [11]. Two studies applied the odds ratio as a measure of association, ranging from 1 [1] [81] to 1.60 [1.31, 1.96] [63]. Only one study used the relative risk and identified an increase from 1.70 [1.51–1.92] among those aged 65 to 74 years with 2CC to 2.31 [1.92–2.77] among those in the same age group with ≥3CC [66].

**Figure 4:**
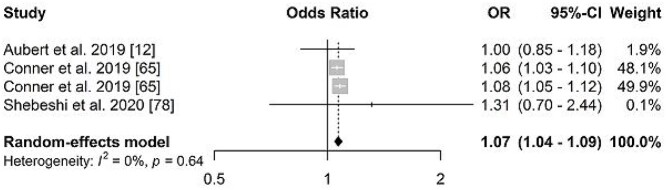
Forest plot of odds ratio of the association between multimorbidity and hospitalization in older adults stratified by sex.

**Figure 5:**
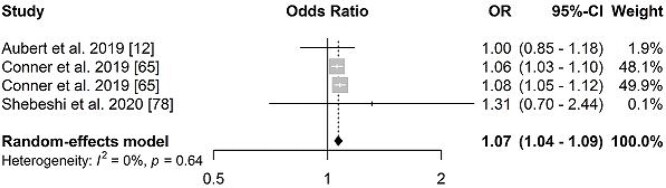
Forest plot of odds ratio of the association between multimorbidity and readmission in older adults.

Multimorbidity was associated with hospitalization in older adults from both genders, OR = 2.10 (95%CI: 1.44; 3.08, *I*^2^: 96%) in women and 1.95 (95%CI: 1.52; 2.49, *I*^2^: 92%) in men (Figure 4). Three studies were included in the meta-analysis on the association between multimorbidity and readmission in older adults (69,82,83), with OR = 1.07 (95%CI: 1.04; 1.09 *I*^2^: 0%) (Figure 5).

## Discussion

To the best of our knowledge, this is the first meta-analysis to assess the impact of multimorbidity on hospitalization and readmission of older adults in high-, middle-, and low-income countries. In this systematic review and meta-analysis, we found a 2.5-fold positive risk of association between multimorbidity and hospitalization, and this risk was similar in studies that analysed ≥2 and ≥3 morbidities, regardless of income level and gender. Multimorbidity increased the risk of hospital readmissions. The studies that analysed length-of-stay associated with multimorbidity did not use measures of association, making it impossible to perform a meta-analysis.

We found that in high-, middle- and low-income countries there was a positive association between hospitalization and multimorbidity with a 2.5 times higher risk. Previous systematic reviews also identified an association between multimorbidity and hospitalization in older adults [3] and use and cost of health services in older adults with multiple chronic conditions [8]. However, they [3, 8] included only studies from high-income countries. A report conducted by the Academy of Medical Sciences of the United Kingdom in 2018 observed that the increase in the number of chronic conditions is associated with hospitalization in low- and middle-income countries; nonetheless, there were still few studies in these countries when compared to those of high income [14].

International reports and guidelines have discussed interventions on how to manage multimorbidity in older adults, such as the World Health Organization guideline [83] and the Academy of Medical Sciences report [14]. According to WHO, for the safe and effective management of multimorbidity is necessary a primary care system that provides comprehensive and integrating care performed by the same team, considering multiple chronic conditions. In this sense, the guideline discusses the importance of training health professionals through the implementation of the multimorbidity theme at undergraduate and graduate levels. This will improve and avoid fragmentation of health care for older adults with multimorbidity [83]. The Academy of Medical Sciences report raised important concerns about the fact that health systems and guidelines are directed to the treatment of a single chronic disease and provide recommendations for their use. It demonstrates that there is a lack of preventive strategies specifically for the management and treatment of older adults with multimorbidity [14].

Most studies (70%) included in this systematic review were conducted in high-income countries, which reflects the lower support and funding for research in middle- and low-income countries, such as Brazil, where the government does not currently support or encourage science [84]. It is also important to acknowledge that studies on multimorbidity and hospitalization are increasingly relevant both due to population ageing and the advent of the COVID-19 pandemic, which increases hospitalization and mortality in those with multimorbidity [85, 86].

Our study has shown that both definitions of multimorbidity, i.e. ≥ 2 and ≥ 3 morbidities, were associated with hospitalization in older adults. Another systematic review, despite not having used the same definitions of multimorbidity as the ones used in our study, found that as the number of diseases in the same individual increased, the probability of hospitalization also increased [8]. This finding highlights the importance of health professionals being prepared to provide integrated care to older adults with multimorbidity to mitigate its adverse health outcomes.

Only 31% of the included studied presented information on the association between multimorbidity and hospitalization by gender. This association was positive regardless of gender and without significant variation between men and women. A previous systematic review that included 35 studies on the association between use and costs of health services among older people with multiple chronic conditions did not find an association between gender and hospitalization [8]. We observed that, despite the prevalence of multimorbidity in older adults being higher in women [3, 23], the occurrence of hospitalization due to multimorbidity was not different between genders. Due to the small number of studies stratified by gender in this meta-analysis, these findings should be interpreted with caution.

One of the objectives of our study was to verify whether the association between multimorbidity and hospitalization changed by age given the high prevalence of multimorbidity in older adults, especially in those aged 85 and older [26]. However, due to the heterogeneity of age groups included in the studies, it was not possible to perform a meta-analysis.

Multimorbidity increased hospital readmission in older adults by 1.07 times. These results corroborate the findings of another systematic review that identified that multiple chronic conditions increase the probability of unplanned hospital readmissions [8]. Although in this meta-analysis we observed a positive association between readmission and multimorbidity, this information should be interpreted cautiously due to the number of studies. In addition, methodological differences regarding the definition of readmission in the studies may have also interfered in the results.

Length of stay was one of the outcomes analysed in this review, but it was observed in only seven of the 33 studies included. The studies on length of stay did not present measures of association with multimorbidity in older adults, only means and standard deviations. Therefore, it was not possible to assess the association with multimorbidity. In addition, some studies stratified this association by age group [55, 63, 66] and different definitions of multimorbidity (≥2 or ≥3 chronic conditions) [11, 55, 61, 81].

Cardiometabolic diseases and cancer were the most used conditions. The heterogeneity related to diseases used to measure and assess multimorbidity is widely discussed by the scientific community [87]. This highlights the lack of consensus on the pre-defined criteria for choosing the list of diseases since some are selected based on data availability [88], prevalence [89] and other reasons. With regard to the data source, self-report was the most used method, which could be explained by the fact that population-based samples tend to be large and/or when other data sources are not available [22].

A few limitations should be acknowledged. First, the impossibility of performing a meta-analysis for several outcomes analysed (readmission and length of stay). Second, the heterogeneity of the results found. Thus, some findings need to be interpreted with caution given the low number of studies found.

The strengths of this systematic review are the use of scales to verify the methodological quality and risk of bias of the included studies and absence of language and year of publication restrictions, broad list of descriptors and databases used, as well as the performance of a meta-analysis. Other positive aspects of our review were: conducting a supplementary search, tracking citations in the reference list of the included studies and relevant systematic reviews, and Google Scholar searches; using two definitions of multimorbidity (i.e. ≥2 and/or ≥3 chronic conditions), making the study broader; and using the age range ≥60 years to include both definitions from developed and developing countries.

## Directions for future studies

As most studies included were from high-income countries, further studies in low- and middle-income countries should be prioritized, especially among women [14, 23]. In view of the high prevalence of multimorbidity worldwide and its impact on hospitalization, further studies are needed to assist in the construction of public policies for better prevention, treatment and continuity of care. We also reinforce the importance of stratification by gender in studies on multimorbidity and hospitalization. Future research should also evaluate the impact of multimorbidity on hospitalization, readmission and length of stay in older adults, as this information is relevant for planning health services and care in the prevention of diseases in this population, as well as economic costs with health. This systematic review evaluates the impact of multimorbidity on hospitalization, readmission and length of stay through a simple disease count. Future research should explore these associations by applying other measures available to assess multimorbidity, such as specific disease clusters or patterns.

## Conclusion

In this systematic review and meta-analysis, multimorbidity (≥2 and ≥3 morbidities) was associated with a higher risk of hospitalization, regardless of the country’s income level and patients’ gender. The occurrence of multimorbidity was associated with higher hospital readmission in older adults. It was not possible to verify if there was an association between multimorbidity and length of stay.

## Supplementary Material

aa-21-2151-File001_afac155
