# Supplementary material for: Association between multimorbidity and hospitalization in older adults: systematic review and meta-analysis
Source: Age Ageing. 2022 Jul 23;51(7):afac155. doi: 10.1093/ageing/afac155 (PMC9308991; doi:10.1093/ageing/afac155)
Supplement: aa-21-2151-File001_afac155 [file aa-21-2151-file001_afac155.docx]

**Association between multimorbidity and hospitalization in older adults: Systematic review and meta-analysis**

**SUPPLEMENTARY DATA**

**Appendix 1:** Methodological quality assessment and strength of evidence

| **Study (year)** | **Study Design** | **Conflict of Interests** | **Ethical  Approval** | **Downs and Black checklist** | | | | | | | | | | | | | | | | | | **GRADE** |
| --- | --- | --- | --- | --- | --- | --- | --- | --- | --- | --- | --- | --- | --- | --- | --- | --- | --- | --- | --- | --- | --- | --- |
|  |  |  |  | **A** | **B** | **C** | **D** | **E** | **F** | **G** | **H** | **I** | **J** | **K** | **L** | **M** | **N** | **O** | **P** | Total | Score# |  |
| Aubert et al. (2019) | cohort | No | No | 1 | 1 | 1 | 2 | 1 | 1 | 0 | 0 | 1 | 0 | 1 | 1 | 1 | 1 | 1 | 0 | 13 | 76% | 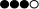 |
| Aubert et al. (2019)  Switzerland/USA/Israel | cohort | No | Yes | 1 | 1 | 1 | 0 | 1 | 1 | 0 | 0 | 1 | 0 | 1 | 1 | 1 | 1 | 1 | 0 | 11 | 64% | 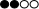 |
| Bähler et al. (2015) | cross-sectional | No | Yes | 1 | 1 | 1 | 2 | 1 | 1 | — | 0 | 1 | 1 | — | 1 | 1 | — | 1 | — | 12 | 92% | 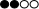 |
| Buja et al. (2020) | cohort | No | * | 1 | 1 | 0 | 1 | 1 | 1 | 0 | 0 | 1 | 1 | 1 | 1 | 1 | 1 | 1 | 0 | 12 | 70% | 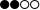 |
| Conner et al. (2019) | cross-sectional | No | * | 1 | 1 | 1 | 0 | 1 | 1 | — | 1 | 1 | 1 | — | 1 | 1 | — | 0 | — | 10 | 76% | 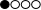 |
| Chamberlain et al. (2019) | cohort | No | Yes | 1 | 1 | 1 | 1 | 1 | 1 | 0 | 1 | 1 | 1 | 1 | 1 | 1 | 1 | 1 | 0 | 14 | 82% | 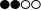 |
| Collerton et al. (2016) | cohort | Yes | Yes | 1 | 1 | 1 | 0 | 1 | 1 | 0 | 1 | 1 | 1 | 1 | 0 | 1 | 1 | 1 | 0 | 12 | 70% | 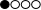 |
| Cheung et al. (2019) | cross-sectional | No | Yes | 1 | 1 | 1 | 1 | 1 | 1 | — | 1 | 1 | 1 | — | 1 | 1 | — | 1 | — | 12 | 92% | 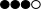 |
| Ensrud et al. (2018) | cohort | No | Yes | 1 | 1 | 1 | 1 | 1 | 1 | 1 | 1 | 1 | 1 | 1 | 1 | 1 | 1 | 1 | 0 | 15 | 88% | 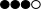 |
| Gandhi et al. (2018) | cross-sectional | No | Yes | 1 | 1 | 1 | 1 | 1 | 1 | — | 1 | 1 | 1 | — | 1 | 1 | — | 1 | — | 12 | 92% | 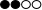 |
| Garcia-Ramirez et al. (2020) | cross-sectional | No | Yes | 1 | 1 | 1 | 0 | 1 | 1 | — | 1 | 1 | 1 | — | 1 | 1 | — | 0 | — | 10 | 76% | 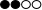 |
| Glynn et al. (2011) | cross-sectional | Yes | Yes | 1 | 1 | 1 | 1 | 1 | 1 | — | 0 | 1 | 1 | — | 1 | 1 | — | 1 | — | 11 | 84% | 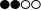 |
| Gruneir et al. (2016) | cohort | No | Yes | 1 | 1 | 1 | 2 | 1 | 1 | 1 | 0 | 1 | 1 | 1 | 1 | 1 | 1 | 1 | 0 | 15 | 88% | 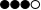 |
| Halonen et al. (2019) | cohort | No | Yes | 1 | 1 | 1 | 1 | 1 | 1 | 1 | 0 | 1 | 0 | 1 | 1 | 1 | 1 | 1 | 1 | 14 | 82% | 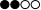 |
| Kim et al. (2020) | cross-sectional | No | * | 1 | 1 | 1 | 0 | 1 | 1 | — | 1 | 1 | 1 | — | 1 | 1 | — | 0 | — | 10 | 77% | 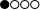 |
| Lai et al. (2019) | cohort | No | * | 1 | 1 | 1 | 2 | 1 | 1 | 0 | 1 | 1 | 1 | 1 | 1 | 1 | 1 | 1 | 0 | 15 | 88% | 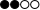 |
| Li et al. (2020) | cross-sectional | No | * | 1 | 1 | 1 | 1 | 1 | 1 | — | 1 | 1 | 1 | — | 1 | 1 | — | 1 | — | 12 | 92% | 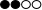 |
| Lochner et al. (2013) | cross-sectional | No | * | 1 | 1 | 1 | 0 | 1 | 0 | — | 0 | 1 | 1 | — | 0 | 1 | — | 0 | — | 7 | 53% | 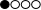 |
| Marthias et al. 2021 | cross-sectional | No | Yes | 1 | 1 | 1 | 1 | 0 | 1 | — | 1 | 1 | 1 | — | 1 | 1 | — | 0 | — | 10 | 76% | 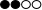 |
| Mini et al. (2017) | cross-sectional | No | Yes | 1 | 1 | 1 | 1 | 1 | 1 | — | 0 | 1 | 1 | — | 1 | 1 | — | 1 | — | 11 | 84% | 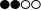 |
| Mitsutake et al. (2019) | cross-sectional | * | Yes | 1 | 1 | 1 | 1 | 1 | 1 | — | 1 | 1 | 1 | — | 1 | 1 | — | 1 | — | 12 | 92% | 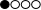 |
| Nägga et al. (2012) | cross-sectional | No | Yes | 1 | 1 | 1 | 0 | 1 | 1 | — | 1 | 1 | 1 | — | 1 | 1 | — | 0 | — | 10 | 76% | 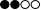 |
| Navickas et al. (2015) | cohort | No | * | 1 | 1 | 1 | 0 | 1 | 1 | 0 | 1 | 1 | 1 | 1 | 1 | 1 | 1 | 0 | 0 | 12 | 70% | 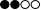 |
| Nunes et al. (2017) | cross-sectional | No | Yes | 1 | 1 | 1 | 1 | 1 | 1 | — | 1 | 1 | 1 | — | 1 | 1 | — | 1 | — | 12 | 92% | 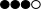 |
| Nunes et al. (2015) | cross-sectional | No | Yes | 1 | 1 | 1 | 0 | 1 | 1 | — | 0 | 1 | 1 | — | 1 | 1 | — | 0 | — | 9 | 69% | 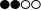 |
| Pati et al. (2020) | cross-sectional | No | Yes | 1 | 1 | 1 | 2 | 1 | 1 | — | 1 | 1 | 1 | — | 1 | 1 | — | 1 | — | 13 | 100% | 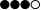 |
| Picco et al. (2016) | cross-sectional | No | Yes | 1 | 1 | 1 | 1 | 1 | 1 | — | 1 | 1 | 1 | — | 1 | 1 | — | 1 | — | 12 | 92% | 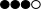 |
| Rodrigues et al. (2018) | cross-sectional | No | Yes | 1 | 1 | 1 | 1 | 1 | 1 | — | 0 | 1 | 1 | — | 1 | 1 | — | 1 | — | 11 | 84% | 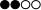 |
| Shebeshi et al. (2020) | cohort | No | Yes | 1 | 1 | 1 | 1 | 1 | 1 | 1 | 1 | 1 | 1 | 1 | 1 | 1 | 1 | 1 | 1 | 16 | 94% | 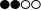 |
| Wagner et al. (2019) | cohort | No | Yes | 1 | 1 | 1 | 2 | 1 | 1 | 0 | 1 | 1 | 1 | 1 | 1 | 1 | 1 | 1 | 0 | 15 | 88% | 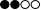 |
| Wang et al. (2018) | cross-sectional | No | Yes | 1 | 1 | 1 | 0 | 1 | 1 | — | 1 | 1 | 1 | — | 1 | 1 | — | 0 | — | 10 | 76% | 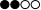 |
| Wolff et al. (2015) | cross-sectional | * | * | 1 | 1 | 1 | 1 | 1 | 1 | — | 0 | 1 | 1 | — | 1 | 1 | — | 1 | — | 11 | 84% | 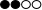 |
| Wister et al. (2016) | cross-sectional | No | Yes | 1 | 1 | 1 | 2 | 1 | 1 | — | 1 | 1 | 1 | — | 1 | 1 | — | 1 | — | 13 | 100% | 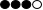 |
| Downs and Black checklist: A) objective clearly stated; B) main outcomes clearly described; C) sample characteristics clearly defined; D) distribution of principal confounders clearly described; E) main findings clearly defined; F) random variability in estimates provided; G) lost to follow-up described; H) probability values reported; I) sample target representative of population; J) sample recruitment representative of population; K) analyses adjusted for different follow-up duration ; L) statistical tests appropriately used; M) primary outcomes valid/reliable; N) sample recruited from the same population; O) adequate adjustment for confounding; and P) losses of sample to follow-up taken into account (corresponding to questions 1-3, 5-7, 9-12, 17,18, 20, 21, 25,26).  Questions G and P were applied only for longitudinal studies. Questions K and N were applied only for case-control and longitudinal studies.  # Score reaches 100% with 13, 15, and 17 points for cross-sectional, case-control, and longitudinal studies, respectively. *, not reported, -, not applied  GRADE, Grading of Recommendations, Assessment, Development and Evaluations; one filled circle, very low quality; two filled circles, low quality; three filled circles, moderate quality; four filled circles, high quality | | | | | | | | | | | | | | | | | | | | | | |

**Appendix 2.** Funnel plot general analysis


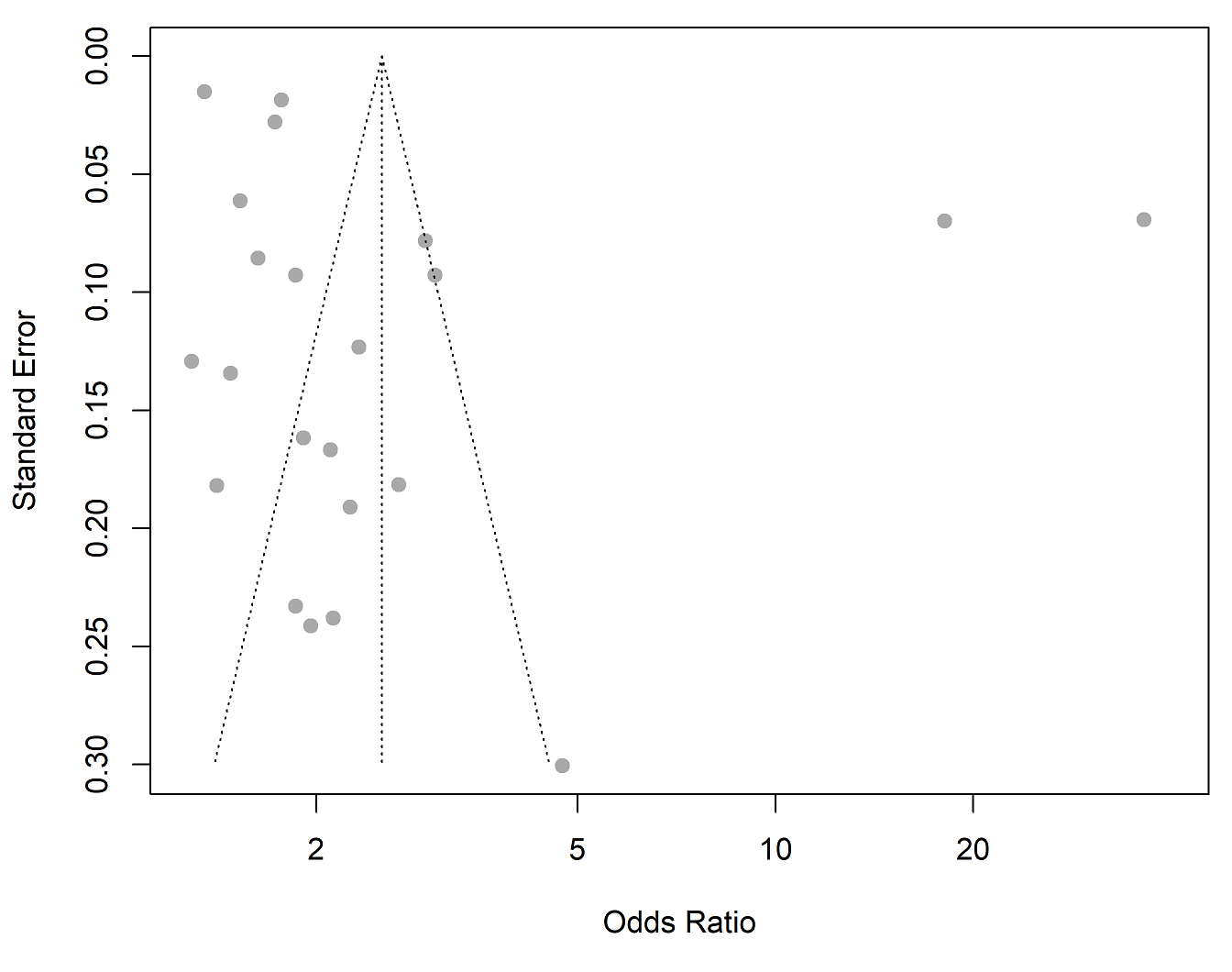


**Appendix 3.** Overview of multimorbidity impact on hospitalizations

**
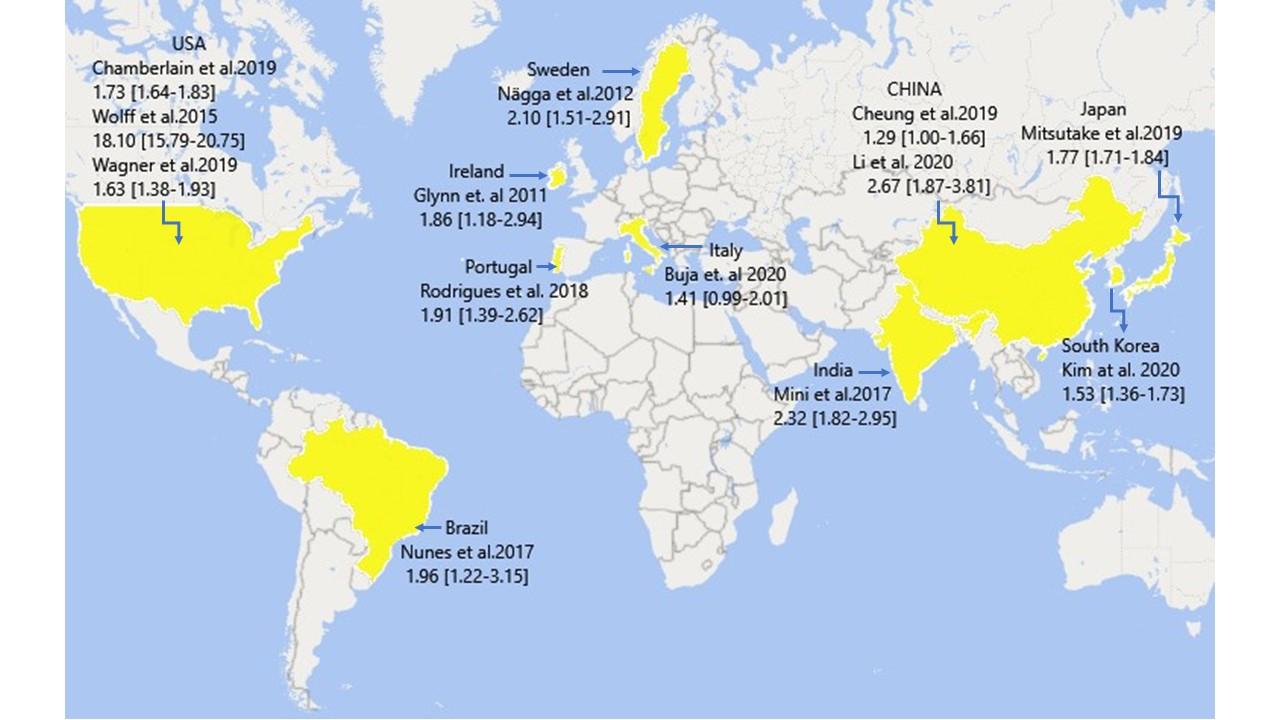
**

**Table S1.** Search strategy for studies on multimorbidity and hospitalization in the older adults.

| 1 = "multiple chronic conditions" OR multimorbidity  OR multimorbidit* OR   "multi morbidit*" OR multi-morbidity   OR "chronic conditions multiple" OR "multiple chronic health conditions" OR  "multiple chronic medical conditions" OR "multiple chronic illnesses" OR "chronic illnesses multiple" OR "multiple chronic diseases" OR multidisease OR multidiseases  OR "multiple condition" OR "complex needs" OR "concurrent chronic conditions" OR "concurrent chronic diseases" OR "concurrent chronic disorders" OR "concurrent chronic health conditions" OR "concurrent chronic illnesses" OR "concurrent chronic medical conditions" OR "multiple chronic disorders" OR "simultaneous chronic illnesses" OR "simultaneous chronic medical conditions" |
| --- |
| 2 = elderly OR elder OR aged OR ageing OR aging OR "old adults" OR "older adults" OR "older people" OR "old people" OR "geriatric" OR "aged patient" OR "aged people" OR "aged person" OR "aged subject" OR "elderly patient" OR "elderly people" OR "elderly person" OR "elderly subject" OR "senior citizen" OR senium |
| 3 = hospitalization OR "patient readmission" OR inpatients  OR hospitalized OR "health services" OR "medical assistance" OR "intensive care unity" OR "health care utilization" OR "length of stay" OR "short stay hospitalization" OR" hospital admission" OR "admission, hospital" OR "patient admission" OR "health care use" OR "health care utilisation" OR "health resource utilization" OR "health service use" OR   "health service utilisation" OR "health service utilization" OR "health service utilization pattern" OR "health services use" OR "health services utilisation" OR "health services utilization" OR "utilization, health care" OR "hospital patient" OR "hospitalised patient" OR "hospitalised patients" OR "hospitalized patient" OR "hospitalized patients" OR "in-hospital patient" OR "in-hospital patients" OR "in-patient" OR "in-patients" OR inpatient OR "patient, hospital" |
| 4 = 1 AND 2 AND 3 |
